# A Variant in the Nicotinic Acetylcholine Receptor Alpha 3 Subunit Gene Is Associated With Hypertension Risks in Hypogonadic Patients

**DOI:** 10.3389/fgene.2020.539862

**Published:** 2020-11-27

**Authors:** Tao Wu, Yujia Wang, Wei Shi, Bi-Qi Zhang, John Raelson, Yu-Mei Yao, Huan-Dong Wu, Zao-Xian Xu, Francois-Christophe Marois-Blanchet, Jonathan Ledoux, Rikard Blunck, Jian-Zhong Sheng, Shen-Jiang Hu, Hongyu Luo, Jiangping Wu

**Affiliations:** ^1^Institute of Cardiology, The First Affiliated Hospital, College of Medicine, Zhejiang University, Hangzhou, China; ^2^Research Centre, Centre Hospitalier de l’Université de Montréal (CHUM), Montreal, QC, Canada; ^3^Children’s Hospital, Zhejiang University School of Medicine, Hangzhou, China; ^4^Department of Cardiology, The Third Affiliated Hospital of Zhejiang Chinese Medical University, Hangzhou, China; ^5^Montreal Heart Institute, Université de Montréal, Montreal, QC, Canada; ^6^Department of Physics, University of Montreal, Montreal, QC, Canada; ^7^Department of Pathology and Physiopathology, College of Medicine, Zhejiang University, Hangzhou, China; ^8^Nephrology Service, Centre Hospitalier de l’Université de Montréal (CHUM), Montreal, QC, Canada

**Keywords:** CHRNA3, acetylcholine receptor, adrenal gland chromaffin cells, single nucleotide variant, hypogonadism, hypertension

## Abstract

*Ephb6* gene knockout causes hypertension in castrated mice. EPHB6 controls catecholamine secretion by adrenal gland chromaffin cells (AGCCs) in a testosterone-dependent way. Nicotinic acetylcholine receptor (nAChR) is a ligand-gated Ca^2+^/Na^+^ channel, and its opening is the first signaling event leading to catecholamine secretion by AGCCs. There is a possibility that nAChR might be involved in EPHB6 signaling, and thus sequence variants of its subunit genes are associated with hypertension risks. CHRNA3 is the major subunit of nAChR used in human and mouse AGCCs. We conducted a human genetic study to assess the association of CHRNA3 variants with hypertension risks in hypogonadic males. The study cohort included 1,500 hypogonadic Chinese males with (750 patients) or without (750 patients) hypertension. The result revealed that SNV *rs3743076* in the fourth intron of *CHRNA3* was significantly associated with hypertension risks in the hypogonadic males. We further showed that EPHB6 physically interacted with CHRNA3 in AGCCs, providing a molecular basis for nAChR being in the EPHB6 signaling pathway.

## Introduction

Erythropoietin-producing hepatocellular kinases (EPHs) comprise the largest family of receptor tyrosine kinases (Eph Nomenclature Committee, 1997). Their ligands are called ephrins (EFNs), which are also cell surface molecules. When EPH and EFN on neighboring cells interact, both molecules are capable of transducing signals into their respective cells. Interactions between EPHs and EFNs are promiscuous. One EPH can interact with multiple EFNs and vice versa. In general, EPHA members bind preferentially to EFNA members, and EPHB members, to EFNB members (Pasquale, 2008).

Erythropoietin-producing hepatocellular kinases and EFNs have functions in many biological systems and processes, such as the nervous (Flanagan and Vanderhaeghen, 1998; Wilkinson, 2000), the vascular (Kuijper et al., 2007), the digestive (Batlle et al., 2002), the immune (Wu and Luo, 2005), and the endocrine systems (Konstantinova et al., 2007). Our lab is the first to report the function of EPHs and EFNs in the immune system (Luo et al., 2001, 2002). We have recently discovered the previously unknown functions of EPHs and EFNs in regulating blood pressure (Luo et al., 2012; Wu et al., 2012; Wang et al., 2015). Our human genetics studies have provided corroborating evidence that five single-nucleotide variants (SNVs) in the EFNB2 (Wang et al., 2016) and two SNVs in the EFNB3 (Tremblay et al., 2017) are significantly associated with human hypertension in a sex-specific manner.

The deletion of EPHB6 results in increased vascular smooth muscle cell (VSMC) contractility but does not affect blood pressure in male *Ephb6* knockout (KO) mice unless the mice are castrated: *Ephb6* KO plus castration leads to increased blood pressure (Luo et al., 2012). We have shown that, in addition to its effects on VSMCs, EPHB6 also regulates catecholamine biosynthesis (Shi et al., 2019) and secretion by adrenal gland chromaffin cells (AGCCs) (Luo et al., 2012). Male *Ephb6* KO mice have reduced 24-hour urine catecholamine levels, counteracting the outcome of increased VSMC contractility, and the sum of these two effects results in normal blood pressure. The castration of male *Ephb6* KO mice leads to a return to normal levels of catecholamine secretion (Luo et al., 2012). This change, concomitantly with enhanced VSMC contractility, results in blood pressure elevation in the castrated *Ephb6* KO mice. These results suggest that EPHB6 and male sex hormones act in concert to regulate catecholamine secretion and blood pressure.

Our further investigation revealed that AGCCs from male *Ephb6* KO mice presented reduced acetylcholine (ACh)-triggered Ca^2+^ influx, depending on the non-genomic effects of testosterone (Wang et al., 2018). We demonstrated that the decreased Ca^2+^ influx resulted from enhanced large-conductance calcium-activated potassium (BK) currents in these cells (Wang et al., 2018).

In addition to the abnormality of BK channels in the *Ephb6*-KO AGCCs, EPHB6 might regulate other ion channels such as the ACh receptor (AChR), whose opening is the first event in triggering catecholamine release in AGCC. There are two types of AChRs, nicotinic AChR (nAChR) and muscarinic AChR, based on their agonists (nicotine and muscarine). nAChR is a ligand-gated ion channel mediating fast responses of ACh (Itier and Bertrand, 2001), while muscarinic AChR (mAChR) is a G-protein-coupled metabotropic receptor with a slower signaling pace (Eglen, 2006).

nAChR is a pentamer with five homomeric or heteromeric subunits, depending on species and cell types. The subunits consist of α, β, δ. ε and γ types. The α type has ten members (α1–10), β type five members (β1–5). In the mouse AGCCs, α1, α3, α4, α7, α9, β1, β2, and β4 are detectable, with α3 and β4 being most abundant (Mousavi and Nordberg, 2006; Wu et al., 2010), similar to that in rat and bovine AGCCs (Campos-Caro et al., 1997; Di Angelantonio et al.,2003). According to subunit-specific blocker/agonists and the measurement of mRNA, α3b4 nAChR is also present and functional in human AGCCs (Mousavi et al., 2001; Perez-Alvarez et al., 2012).

Multiple proteins can interact with nAChR and positively or negatively modulate its function (Jones et al., 2010). EPHB6 might be one such regulator, and nAChR might lie in the EPHB6 signaling pathway explaining its role in blood pressure control. If so, variants in nAChR subunits should be associated with hypertension risks in a testosterone-dependent way, similar to those of EPHB6. In this study, we conducted a human genetic study to assess the association of variants in CHRNA3, the major subunit of nAChR in AGCCs, with hypertension risks in hypogonadic, hypertensive patients. We also investigated the possible physical interaction of EPHB6 with nAChR.

## Materials and Methods

### Patient Population

The details of the patient population were previously described (Wu et al., 2018) but are presented here again for the convenience of readers. A total of 4,480 male patients ≥40 years old from the Cardiology Ward, Endocrinology Ward, and Physical Examination Center of First Affiliated Hospital, College of Medicine, Zhejiang University in Hangzhou, China, were recruited for this study. They were tested for total plasma testosterone levels. Those with hypogonadism [plasma total testosterone levels <346 ng/dL, the International Society’s cut-off level recommended for the Study of the Aging Male (Traish et al., 2011)] were retained. Among those hypogonadic individuals, 982 were diagnosed with primary hypertension and were considered as cases. The primary hypertension phenotype was defined as having recorded systolic pressure >140 mm Hg or diastolic pressure >90 mmHg or having been actively treated for hypertension, excluding known conditions or medication use that could cause BP to increase. All other medical conditions were allowed as long as they were not likely to cause hypertension. All types of medication in the last 3 months were allowed except those that are known to affect testosterone levels, such as testosterone replacement therapy. Seven hundred and eighty-eight normotensive hypogonadic patients among the cohort were retained as controls. Seven hundred and fifty cases and 750 controls were selected with an attempt to match their ages as closely as possible. The systolic and diastolic BP and the values of potential covariate parameters (i.e., age, plasma testosterone level, heart rates, body mass index (BMI), serum uric acid levels, and smoking status), which could be implicated hypertension risks, of the case and control groups are shown in Supplementary Tables 1, 2.

### Blood Sample Collection and Plasma Total Testosterone Measurements

Venous blood samples were drawn from all subjects after an overnight fast of at least 8 h. Five mL of blood was collected into vacuum tubes with the anticoagulant EDTA-K^+^ and centrifuged at the collection site within 1 h. Cell pellets were frozen until DNA extraction.

Plasma total testosterone levels were measured with Siemens Immulite 2000 Total Testosterone Kits on Siemens Immulite 2000 Immunoassay Analyzer according to the manufacturer’s protocols.

### DNA Extraction and Purification

Sample DNA was extracted using DNeasy Blood and Tissue Kit (Cat. 69506, QIAGEN, Hilden, Germany) according to the manufacturer’s instructions. Purified DNA quantity and quality were assessed by Qubit^®^2.0 Fluorometer (Q32866, Invitrogen, Carlsbad, CA, United States) and 1% agarose gel electrophoresis. Samples with DNA quantity ≥ 2 μg and optical density (OD) 260 nm/280 nm = 1.8–2.0 were submitted to SNV assay.

### Candidate Tag Single Nucleotide Variants (SNVs)

Tag SNVs were chosen from the chromosomal regions at 15q25.1 between positions 78,837,801 and 78,959,037 (Build 37/hg 19), containing the *CHRNA3* gene and 50 kb on either side of the gene, using the Tagger Program (De Bakker et al., 2005) with Han Chinese in Beijing (CHB) linkage-disequilibrium (LD) data. Tag SNVs were chosen with minimum *r*^2^ > 0.80 and minor allele frequency >0.05. Candidate tag SNVs were then submitted for analysis by Illumina Software for compatibility with the GoldenGate multiplexing process. Alternative Tag SNVs were chosen for those Tag SNVs determined to be incompatible with the multiplexing technology, and the new Tag SNVs were then re-submitted for a new Illumina software analysis to determine the compatibility of the new set of SNVs including previously compatible SNVs and the new alternative Tag SNVs. This process continued iteratively until all Tag SNVs were found to be compatible for use by the multiplexing technology. Ultimately, 14 tag SNVs were chosen for this region. The *Bonferroni*-corrected critical *p*-values (*Pcrit* = 0.05/14 = 0.0037) were calculated for SNVs with the hypertension association test, assuming tag SNVs represent independent statistical tests, when performing association analysis across the region.

### Tag SNV Genotyping

The tag SNVs were genotyped by the Shanghai Biotechnology Corporation using the Illumina GoldenGate genotyping platform according to the manufacturer’s instructions. Those SNVs with a call rate of less than 90% were filtered out and not analyzed.

### Association Analysis

The genotyped SNVs were tested for Hardy-Weinberg equilibrium and were analyzed for association with hypertensive versus normotensive status using the PLINK program (Purcell et al., 2007) and a logistic regression model both with and without covariates, the choice of which is described in the Results section. Each SNV was assigned a reference allele and an alternative allele, the SNV of the reference allele being given a numeric code of 1, and the alternative allele, 0. The sum of the numeric codes of the whole reference allele in each individual was used as the genotype value. The sums of the genotype values of all cases and controls were calculated, respectively, and were entered into the logistic regression equation as the genotype terms for each group. This approach is analogous to an additive genetic model.

### Reverse Transcription-Quantitative Polymerase Chain Reaction (RT-qPCR)

mRNA levels of *Chrna3* and *Chrnb4* in the mouse adrenal glands were measured by RT-qPCR. Total RNA from adrenal glands of male *Ephb6* KO and WT mice was extracted with TRIzol^®^ (Invitrogen, Burlington, ON, Canada) and reverse-transcribed with iScript^TM^ cDNA Synthesis Kit (Bio-Rad Laboratories (Canada) Ltd., Mississauga, ON, Canada). The sequences of qPCR primers used were listed in Table 1. The qPCR condition was as follows: 2 min at 50°C, 2 min at 95°C, followed by 40 cycles of 10 s at 94°C, 20 s at 58°C, and 20 s at 72°C. β-actin mRNA levels were used as internal controls. Data from 20 to 30 cycles of amplification were used. Samples were assayed in duplicate. Data were expressed as signal ratios of target RNA/β-actin mRNA.

**TABLE 1:** RT-qPCR primer sequences.

Gene	Sense sequences	Antisense sequences
*b-actin*	5′-TCGTACCACAGGCATTGTGATGGA-3′	5′-TGATGTCACGCACGATTTCCCTCT-3′
*Chrna3*	5′-CTGGTGAAGGTGGATGAAGTAA-3′	5′-GGTAGTCAGAGGGTTTCCATTT-3′
*Chrnb4*	5′-CTGGGTTGTAGTGGGATGATATG-3′	5′-GGCTGACTGCCAATAGTCTTAG-3′

### Primary AGCC Culture

The adrenal glands from 8- to 10-week old mice were isolated, and the fat and the cortex were removed from these glands. Papain (P4762, Sigma-Aldrich, Oakville, ON, Canada) was activated with 5 mM L-cysteine. Adrenal gland medullae were digested with papain in Hank’s buffer (2 medullae/100 ml Hank’s buffer containing 4 units of activated papain) at 37°C for 25 min. They were washed twice with Hank’s buffer and then triturated by pipetting in 300 ml Hank’s buffer until they became feather-like. Cells were pelleted at 3,700 *g* for 3 min and resuspended in DMEM containing 15% FCS for culture.

### Immunoprecipitation and Immunoblotting

HEK293 cells were co-transfected with plasmid pCEP4-HA-m*Ephb6* (expressing the HA-tagged mouse EPHB6 intracellular domain (aa 2,268 to 3,537)) and plasmid MR226689 (OriGene, Rockville, MD, United States; expressing Myc-tagged mouse CHRNA3), or control empty pCMV6-Entry Vector (PS100001; OriGene). After 48 h, the cells were lysed using radioimmunoprecipitation assay buffer (RIPA), which contained a cocktail of protease inhibitors (Roche Applied Science, Meylan, France). The lysates were pre-cleared with 30 μl protein G-agarose beads (17061801; GE Healthcare Life Sciences, Mississauga, ON, Canada) and then precipitated with mouse anti-Myc mAb (sc-40; Santa Cruz Biotechnology, Dallas, United States) or normal mouse IgG (sc-2025; Santa Cruz Biotechnology) plus protein G-agarose beads at 4°C with gentle rotation overnight. The protein-bead complexes were mixed with SDS-PAGE-loading buffer and boiled for 5 min. Samples were resolved in 10% SDS-PAGE and transferred to PVDF membranes, which were blotted with rabbit anti-HA mAb (3724; Cell Signaling Technology, Danvers, MA, United States), mouse anti-Myc Ab (sc-40; Santa Cruz Biotechnology), and rabbit anti-b-actin Ab (4967; Cell Signaling Technology). Blots were washed and incubated with fluorophore-conjugated secondary Abs (IRDye^®^680RD-conjugated donkey anti-mouse IgG Ab, P/N 925-68072, LI-COR Biosciences, Lincoln, NE, United States; or IRDye^®^800CW-conjugated donkey anti-rabbit IgG Ab, P/N 925-32213, LI-COR Biosciences). All the Abs were used at the manufacturer’s recommended dilutions. Signals were visualized by the Odyssey infrared imaging system (LI-COR Biosciences).

### Immunofluorescence Microscopy

Adrenal gland chromaffin cells were cultured in 6-well plates with cover glass placed at the bottom of the wells. After one day, the cells were washed once with PBS and fixed with paraformaldehyde (4%) for 20 min. The cells were blocked with 10% FBS in PBS for 20 min and then incubated with goat anti-mouse EPHB6 Ab (AF611; R&D Systems, Minneapolis, MN, United States) and rabbit anti-mouse CHRNA3 Ab (ABN281; Millipore, Etobicoke, ON, Canada) overnight at 4°C. Cells were then reacted with Alexa Fluor-488-conjugated donkey anti-goat IgG Ab (A-11055; Thermo Fisher Scientific, Waltham, MA, United States) for EPHB6 and with rhodamine-conjugated goat anti-rabbit IgG Ab (31670; Thermo Fisher Scientific) for CHRNA3 at room temperature for 2 h. The cells were embedded with ProLong^®^ Gold anti-fade reagent (Invitrogen). Cell staining was examined with a Zeiss microscope.

### Fluorescence Resonance Energy Transfer Microscopy (FRET)

Adrenal gland chromaffin cells were cultured in 6-well plates with cover glass placed at the bottom of the wells. After one day, the cells were washed once with PBS and fixed with paraformaldehyde (4%) for 20 min. The cells were blocked with 10% FBS in PBS for 20 min and then incubated with goat anti-mouse EPHB6 Ab (AF611; R&D Systems, Minneapolis, MN, United States) and rabbit anti-mouse CHRNA3 Ab (ABN281; Millipore, Etobicoke, ON, Canada) overnight at 4°C. The cells were then reacted with Alexa Fluor-488-conjugated donkey anti-goat IgG Ab (A-11055; Thermo Fisher Scientific, Waltham, MA, United States) for EPHB6 and with rhodamine-conjugated goat anti-rabbit IgG Ab (31670; Thermo Fisher Scientific) for CHRNA3 at room temperature for 2 h. The cells were embedded with ProLong^®^ Gold anti-fade reagent (Invitrogen). The FRET signal was examined under a Leica TCS SP5 laser-scanning confocal microscope (Leica Microsystems Inc., Concord, ON, Canada). Rhodamine was the donor fluorophore, and Alexa Fluor-488, the acceptor fluorophore.

Fluorescence resonance energy transfer microscopy was measured with acceptor photobleaching (AB) by FRET AB Wizard software (Leica Microsystems Inc.). All necessary controls for AB, such as cells with relevant single fluorescence staining, were performed to satisfy background deductions in calculating FRET efficiency, as required by the software.

Acceptor photobleaching FRET efficiency was calculated by fluorescence intensity of the donor before (D^*pre*^) and after (D^*post*^) acceptor-selective photobleaching, according to the following formula:

AB FRET efficiency = (D^*post*^ − D^*pre*^) / D^*post*^

## Results

We used 750 hypogonadic men with hypertension as cases and 750 age-matched normotensive hypogonadic men as controls to assess the association of *CHRNA3* variants with hypertension. The mean measured blood pressure (systolic as well as diastolic) was significantly higher in the cases (125.42 + 0.54/76.45 + 0.36 mmHg, mean + SE) than the controls (116.6 + 0.43/72.06 + 0.32 mmHg) (*p* = 2.2 × 10^–16^) for both systolic and diastolic pressures (Supplementary Table 1), but the case blood pressure did not reach the hypertension diagnostic criteria, i.e., systolic pressure >140 mmHg and/or diastolic pressure >90 mmHg. This was due to the fact that the majority of the cases (93.7%) were previously diagnosed and were under anti-hypertension medication, which controlled their measured blood pressure within the normal range.

We performed a covariate analysis to test the effects of non-genetic factors in the determination of case and control status (hypertension) in our sample (Supplementary Table 2). We conducted simple logistic regression analysis on several phenotype candidate covariates that we suspected might differ between cases and controls (variable 1 for cases, 0 for controls). These were age, plasma testosterone levels, heart rates, body mass index (BMI), serum uric acid levels, and smoking status. Each regression was done for a single phenotype covariate value only without any genotype factor included.

Age, heart rate, BMI, and serum uric acid levels were significantly different between cases and controls in the simple regression analyses and were retained for further analysis. In addition, the testosterone level was nearly significant at *p* = 0.05, so it is also included since the assumption of hypogonadism in the whole cohort is central to this analysis.

Realizing that some of these covariates could be correlated among themselves, we attempted to consider their combined effect on case and control status. In order to do so, we performed a multiple logistic regression with all of the retained candidate covariates but without any genotype factor. All of the retained covariates from the simple regression analyses, when combined in a multiple regression model without a genotype factor, remained significantly different between cases and controls, again with the exception of testosterone levels, which were nearly significant (*p* = 0.0506). (Supplementary Table 3). Therefore, we retained age, heart rate, BMI, serum uric acid levels, and blood testosterone levels in the genetic regression models subsequently performed for each SNV separately. All covariate factors were included in each of the genetic multiple regression analysis. There may have been slight differences in covariate measures for each SNV due only to differences in missing values, but this effect will have been slight as the data genotype completeness was controlled and variation kept within strict limits. Essentially, the covariate effects remained constant for all SNV analyses, and the significance of each genotype term was tested once the consistent covariate effects were removed.

**FIGURE 1:**
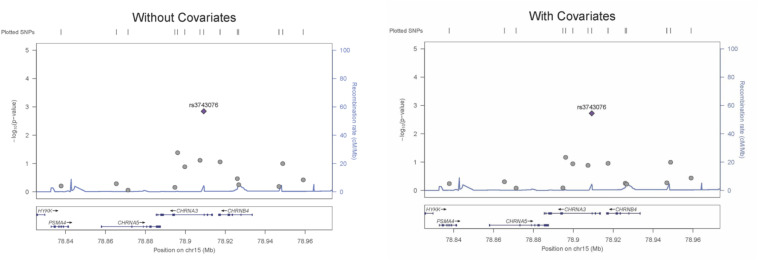
LocusZoom plots for an association of all SNVs assayed across the CHRNA3 gene region for regression models with and without covariates. Left *Y*-axis: –Log_10_ (*p*-value) of the SNV association with hypertension. Right *Y*-axis: recombination rate (cM/Mb). *X*-axis: the position of SNVs and the *CHRNA3* gene on chromosome 15.

The genetic association was tested for all SNVs using logistic regression models, both with and without covariates. SNV *rs3743706* (chromosome 15, position 78,909,227; build 37/hg 19), which is intronic to *CHRNA3* lying 138 bp 5′ of exon 5, was significant at *p*-values below the critical multiple testing *p*-value *(p-crit)* of 0.0037 for models both with and without covariates (*p* = 0.00190 and *p* = 0.00143, respectively). Table 2 presents the genotyped tag SNVs along with their positions, reference alleles, minor alleles, minor allele frequencies, odds ratios with respect to the reference alleles, standard errors of those odds ratios, and the *p*-values for the significance of the odds ratios for association in the models with or without covariates. The SNV with a *p*-value below *p*-crit along with its associated parameters is in bold. Figure 1 shows LocusZoom plots illustrating association *p*-values for all the 14 SNVs calculated without or with covariates, with *rs3743706* showing the highest −log_10_ *p*-value. The plots also illustrate the location of this significant SNV in the *CHRNA3* and the recombination rate in the analyzed region. Analysis with covariates did not reduce the *p*-value of *rs3743706*, compared to the analysis without covariates, suggesting that this SNV’s association with hypertension is independent of the phenotypes used in the logistic multiple regression model.

**TABLE 2:** Logistic regression association tests for SNVs in the CHRNA3 gene region with and without covariates.

Marker						Model with Covariates			Model without Covariates		
SNV	Position (Build 7/hg 19)	Reference Allele	Alternate Allele	Minor Allele	Minor Allele Frequency	Odds Ratio	Standard Error of Odds Ratio	*p*-value	Odds Ratio	Standard Error of Odds Ratio	*p*-value
rs4887062	78837801	G	A	G	0.193	1.052	0.091	0.57860	1.045	0.090	0.62210
rs588765	78865425	T	C	T	0.469	1.082	0.115	0.49470	1.076	0.113	0.51810
rs680244	78871288	T	C	T	0.492	1.025	0.115	0.82760	1.018	0.113	0.87200
rs3743078	78894759	C	G	C	0.229	0.980	0.088	0.81740	0.967	0.087	0.69600
rs1317286	78896129	A	G	G	0.105	1.246	0.121	0.06814	1.271	0.118	0.04167
rs11637630	78899719	G	A	G	0.448	0.891	0.073	0.11500	0.897	0.072	0.13090
rs2869546	78907345	C	T	C	0.195	1.196	0.118	0.13100	1.229	0.117	0.07733
**rs3743076**	**78909227**	**T**	**A**	**T**	**0.257**	**1.307**	**0.086**	**0.00190**	**1.311**	**0.085**	**0.00143**
rs1948	78917399	A	G	A	0.471	1.125	0.074	0.11090	1.132	0.073	0.08789
rs950776	78926018	T	C	C	0.266	0.942	0.103	0.56150	0.908	0.102	0.34360
rs12440014	78926726	C	G	G	0.454	1.039	0.073	0.60040	1.043	0.072	0.56370
rs1021070	78946863	C	G	C	0.148	0.939	0.103	0.54010	0.956	0.101	0.65100
rs7166158	78948753	T	A	T	0.228	1.155	0.088	0.10160	1.153	0.087	0.10100
rs12594550	78959037	C	G	C	0.213	0.922	0.091	0.36610	0.926	0.088	0.37930

**FIGURE 2:**
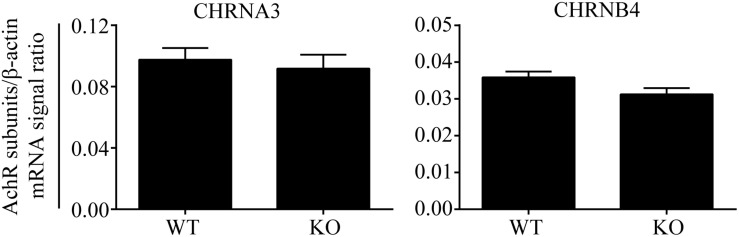
Similar Chrna3 and Chrnb4 mRNA expression levels in AGCCs from WT and EPHB6 KO mice. Mouse *Chrna3* and *Chrnb4* mRNA levels were measured by RT-qPCR, using total RNA from adrenal glands of male *Ephb6* KO and WT mice. β-actin mRNA levels were employed as internal controls. Each sample was analyzed in duplicate. Three independent experiments were conducted, and the pooled data of these experiments were expressed as signal ratios (means ± SE) of target R.N.A./β-actin mRNA. There was no significant difference between WT and *Ephb6* KO AGCCs in their *Chrna3* and *Chrnb4* mRNA levels (two-way paired Student’s *t*-test).

The results of the human genetic study were compatible with our hypothesis that AChR in AGCCs lies within the EPHB6 signaling pathway. If so, EPHB6 might also regulate AChR expression or interact with AChR directly to regulate its function. We first investigated whether *Ephb6* KO resulted in altered expression of the major nAChR subunits α3 and β4 in AGCCs. As shown in Figure 2, the mRNA expression of these two major subunits in KO AGCCs from male mice remained unchanged, compared to their wild type (WT) counterparts. This excludes the possibility that EPHB6 regulates AChR function by modulating the expression of its major components α3 and β4 at the gene level and favors the possibility that the putative regulation might occur through EPHB6 and AChR interaction at the protein level.

**FIGURE 3:**
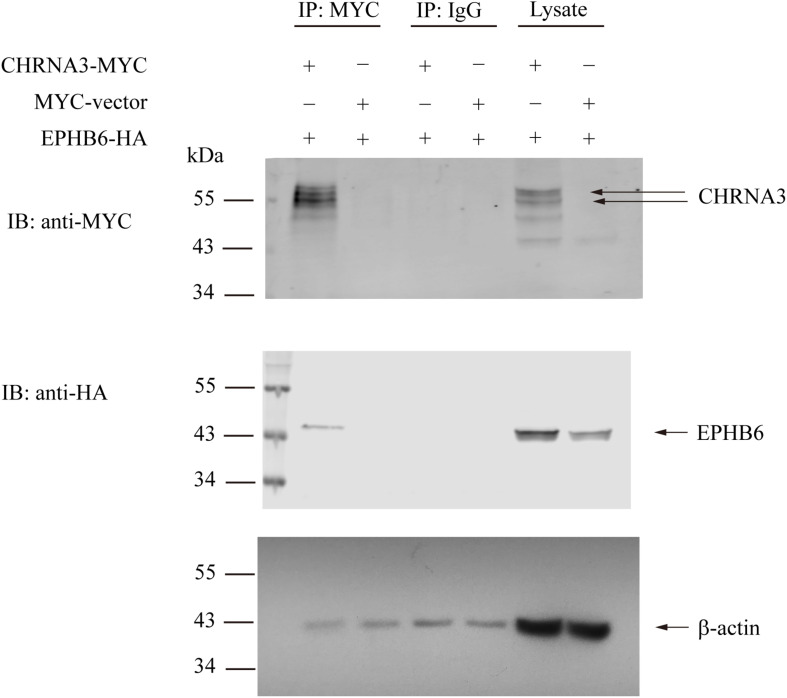
Mouse EPHB6 physically interacts with CHRNA3 according to immunoprecipitation. HEK293 cells were co-transfected with plasmids expressing HA-tagged EPHB6 intracellular domain (aa 2268 to 3537) (EPHB6-HA) and Myc-tagged CHRNA3 (CHRNA-MYC), or an empty control vector (MYC-vector), as indicated. The cell lysates were precipitated with anti-Myc mAb, or normal mouse IgG, as shown. The precipitates were resolved in 10% SDS-PAGE and immunoblotted with anti-Myc (first row) and anti-HA (second row) Abs followed by fluorophore-conjugated secondary Abs. The membranes were also blotted with anti-b-actin Ab (row 3) to show equal loading. Signals were visualized by the Odyssey infrared imaging system (LI-COR Biosciences). The experiments were conducted three times, and representative results are presented. Arrows point to CHRNA3 and EPHB6 bands.

We then examined the possible interaction between EPHB6 and AChR. HEK293 cells were transfected with the HA-tagged mouse EPHB6 intra-cellular domain and Myc-tagged mouse CHRNA3. As shown in Figure 3, EPHB6-HA and CHRNA3-Myc both presented in the lysates of HEK293 cells that were transfected with EPHB6-HA or CHRNA3*-*Myc, but not in cells transfected with the control plasmid. EPHB6-HA was found in the anti-Myc (for CHRNA3-Myc) precipitates (first lane) of cells transfected with both EPHB6-HA and CHRNA3-Myc expressing plasmids, but not in the control precipitates from cells transfected with Myc-vector (second lane). CHRNA3 (the two major bands indicated by arrows) only appeared in lysates and precipitates from cells transfected with CHRNA3-Myc plasmid (lanes 1 and 5). There were some minor non-specific bands observed in anti-My Ab blotting. The abundance of the non-specific proteins was different in the lysates and immunoprecipitates, so the patterns of the non-specific protein bands were different in lanes 1 and 5. Nevertheless, the two CHRNA3 bands were present in both the lysates and immunoprecipitates. Non-specific Ab did not precipitate CHRNA3-Myc or EPHB6-HA (middle two lanes). Beta-actin immunoblotting was used to show similar input of lysates. For immunoprecipitates, the similar intensity of b-actin bands were present in all the lanes, including those controls of normal IgG and empty vectors. The b-actin in the immunoprecipitates was due to non-specific carryover by the beads, but such non-specific carryover indicated that the absence of EPHB6 signals in the control lanes was not due to lack of or unequal protein inputs. This result indicates that EPHB6 physically interacts with AChR via its major subunit CHRNA3. Since the EPHB6 intracellular domain-expressing plasmid was used in this experiment, the results also suggest that the EPHB6 intracellular domain is responsible and sufficient to bind to CHRNA3.

**FIGURE 4:**
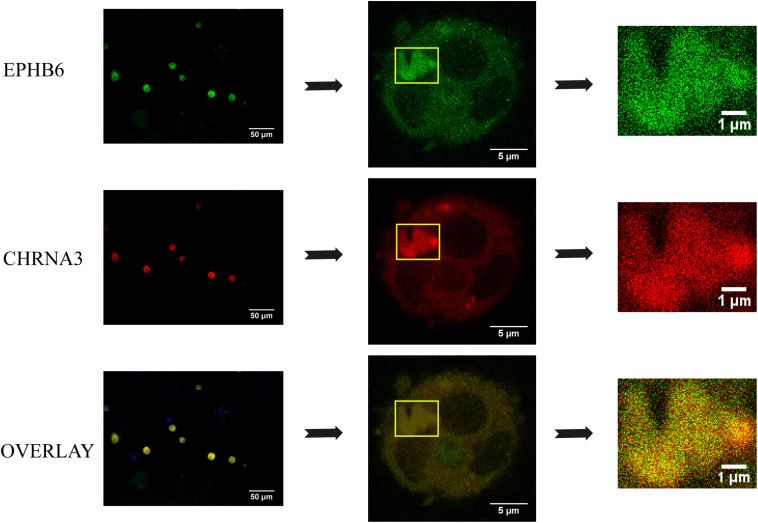
EPHB6 co-localized with CHRNA3 in mouse AGCCs according to confocal microscopy. AGCCs from male WT mice were fixed and stained with anti-EPHB6 and anti-CHRNA3 Abs followed by Alexa Fluor-488-conjugated donkey anti-goat IgG Ab and rhodamine-conjugated goat anti-rabbit IgG Ab, respectively. Three independent experiments were conducted, and micrographs from a representative experiment are shown. The rectangles in the left panels were enlarged at the right. EPHB6 was in pseudo green, and CHRNA3, pseudo red.

To assess the EPHB6 and CHRNA3 interaction in a more physiological setting, we examined EPHB6 and CHRNA3 expression in mouse AGCCs using confocal microscopy and found that these two molecules were colocalized (Figure 4).

**FIGURE 5:**
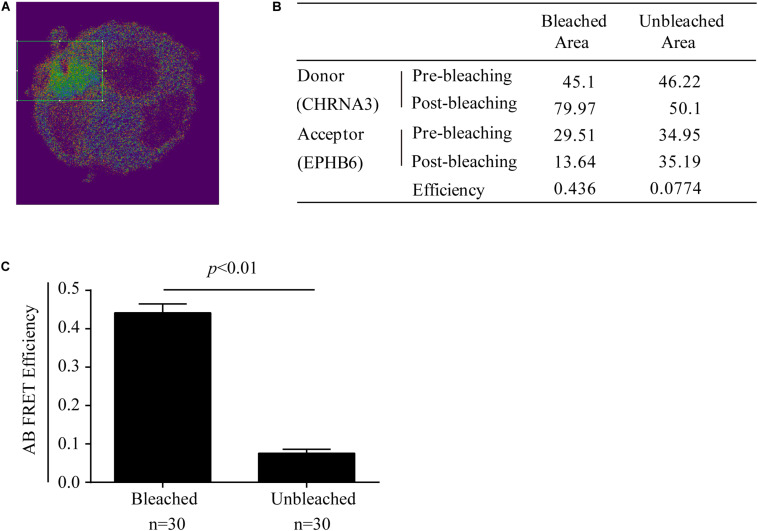
Mouse EPHB6 interacts with CHRNA3 in WT AGCCs according to FRET. FRET was measured with acceptor photobleaching (AB). All the necessary controls for AB, such as cells with relevant single fluorescence staining, were performed to satisfy background deductions in calculating FRET efficiency. AB FRET efficiency was calculated by fluorescence intensity of the donor before (D^*pre*^) and after (D^*post*^) acceptor-selective photobleaching, according to the following formula: AB FRET efficiency = (D^*post*^ – D^*pre*^) / D^*post*^. **(A)** Merged image of an EPHB6-(pseudo-green) and CHRNA3-(pseudo-red) stained AGCC from a WT male mouse. The rectangle indicates the bleached area. **(B)** Donor and acceptor signal measurements and AB FRET efficiency of a representative AGCC from a WT male mouse. **(C)** AB FRET efficiency of bleached and unbleached areas. Data of 7–10 AGCCs from 3 independent experiments were pooled and presented with means ± SE. The differences between the AB FRET efficiency values of bleached areas and unbleached areas were highly significant (*p* < 0.01; Two-way Student’s *t*-test).

The possible physical interaction between EPHB6 and CHRNA3 in WT mouse AGCCs was further examined using a different approach: acceptor photobleaching (AB) FRET. During AB, fluorescence from the acceptor EPHB6 was selectively depleted without influencing donor CHRNA3 signals. The donor and acceptor fluorescence intensity before and after photobleaching in the bleached region (rectangles in Figure 5A) and the unbleached region of AGCCs were quantified. Data from a representative cell are listed in the inset table (Figure 5B). After AB, the signal intensity of donor CHRNA3 was significantly increased in the bleached but not in the unbleached area, compared to that observed before photobleaching. This indicates that donor fluorophore energy could not be transferred to the acceptor due to the acceptor fluorophore bleaching, demonstrating that the donor and acceptor existed in proximity. The acceptor fluorophore intensity was reduced in the photobleached but not unbleached area, again as expected. FRET efficiency from CHRNA3 to EPHB6 of areas inside and outside the bleached regions for more than 7–10 randomly selected cells from 3 independent experiments is shown in Figure 5C. The results show that CHRNA3 and EPHB6 in mouse AGCCs are in very close proximity of <10 nm, which is conventionally considered as a direct association.

## Discussion

We previously demonstrated that EPHB6 and testosterone jointly regulated adrenal gland catecholamine secretion and blood pressure in mice (Luo et al., 2012). The major event triggering catecholamine secretion in AGCCs from the male EPHB6 KO, Ca^2+^ influx, was compromised, and this was accompanied by decreased catecholamine release (Wang et al., 2018). We found that one of the causes for the compromised Ca^2+^ influx was the earlier closure of the BK channel in AGCCs. This premature closure led to quicker AGCC repolarization, hence, to earlier termination of the Ca^2+^ influx and reduced total Ca^2+^ influx (Wang et al., 2018). We wondered whether EPHB6 also affected the Ca^2+^ influx by interacting with other ion channels. nAChR is a ligand-gated ion channel. The opening of nAChR after ligand Ach binding is the first event in AGCC depolarization, which results in the subsequent large Ca^2+^ influx followed by catecholamine secretion. Thus, nAChR in AGCCs is possibly involved in EPHB6 signaling pathways related to blood pressure regulation. This prompted us to conduct a human genetic study to assess the association of variants of a major nAChR subunit CHRNA3 with hypertension risks in hypogonadic male hypertensive patients. These patients had augmented chances of having detrimental variants in the EPHB6 signaling pathways (including AChR subunits) due to their hypogonadic condition because hypertension was only revealed in castrated *Ephb6* KO mice (Luo et al., 2012). Indeed, SNV *rs3743706* within *CHRNA3* was found to be significantly associated with hypertension risks in this cohort.

The significant SNV, rs3743706, is located in the fourth intron of *CHRNA3*. This SNV might and might not be a functional variant. This SNV itself or nearby functionally active variants in linkage disequilibrium (LD) with it in this intron could be part of an intronic enhancer/repressor regulating CHRNA3 expression levels. Such intronic regulators are not uncommon. For example, HCN4 has a potent enhancer in its first intron controlling its expression (Kuratomi et al., 2009). The genomic sequence from 100-bp upstream to 100-bp downstream of this SNV was surveyed for the presence of possible regulatory elements using software available in the UCSC Genome Browser (EPDnew, EPDnewNC, ENCODE Candidate *Cis*-Regulatory Elements (cCREs), GeneHancer, CpG islands, H3K27Ac Mark, ORegAnno, NCBI RefSeq Functional Elements, Double Elite, Hi-C and Micro-C). No conserved enhancer/repressor motifs or transcription factor-binding sites were found in this region (Supplementary Figure 1). We cannot exclude the existence of cryptic and non-canonical regulator elements for which *rs3743706* is a part.

Alternatively, the functional variants could be in LD with this SNV and located in the nearby exons or introns. If so, sequence variants in CHRNA3 protein or enhancer/repressor of its gene might be responsible for the alteration of its function or expression, respectively.

*CHRNA3* SNVs have not been found to be significantly associated with hypertension risks in many genome-wide association studies (Wellcome Trust Case Control Consortium, 2007; Adeyemo et al., 2009; Levy et al., 2009; Org et al., 2009; Hiura et al., 2010; Padmanabhan et al., 2010; International Consortium for Blood Pressure Genome-Wide Association Studies et al., 2011; Slavin et al., 2011; Guo et al., 2012). However, testosterone levels are not stratified in these studies. Since the majority of the males are testosterone sufficient in the general population, *CHRNA3* variants would not be associated with blood pressure in the majority of individuals in these studies, while the smaller subpopulation of patients with both hypogonadism and *CHRNA3* variants would be obscured statistically and so could not reach significance in a GWAS even if they were hypertensive, unless samples were stratified according to testosterone levels.

Our genetic study was performed using an ethnic Han cohort in China. Additional studies using a different Han cohort and a cohort of different genetic backgrounds will be needed to confirm and generalize our conclusion.

There are different views regarding whether catecholamines released from the adrenal glands play a critical role in primary hypertension. The adrenal glands produce all three major species of catecholamines: epinephrine, norepinephrine, and dopamine. However, while plasma epinephrine is mainly derived from the adrenal gland, plasma norepinephrine predominantly comes from sympathetic nerve endings. Dopamine levels in plasma are very low. Some early studies showed that epinephrine levels are associated with hypertension in humans and animal models (Axelrod, 1976; Buhler et al., 1982; Dominiak and Grobecker, 1982; Goldstein, 1983; Borkowski and Quinn, 1984; Jablonskis and Howe, 1994). In recent decades, chronic stress (Sparrenberger et al., 2009; Liu et al., 2017) and systemic low-level sterile inflammation (De Miguel et al., 2015) are positively correlated to primary hypertension. Such conditions are normally associated with elevation of plasma norepinephrine, implicating the involvement of the nervous system, although in many cases, augmentation of epinephrine levels is also observed (; Rivier et al., 1989; Pende et al., 1990; Corssmit et al., 1996; Kannan et al., 1996; Esler et al., 2008a,b; Byrne et al., 2018). In a mouse model with genetic manipulation, TRPM4 deletion resulted in increased catecholamine secretion by AGCCs and hypertension (Mathar et al., 2010). These data collectively suggest that the catecholamine release from the adrenal glands contributes to primary hypertension.

The role of testosterone in blood pressure is controversial. Testosterone increases blood pressure in many animal studies (Masubuchi et al., 1982; Chen and Meng, 1991; Reckelhoff et al., 1998). However, hypogonadism in humans also increases the risk of hypertension (Hughes et al., 1989; Phillips et al., 1993; Jaffe et al., 1996; Liu et al., 2003; Zitzmann, 2009; Garcia-Cruz et al., 2012). In several human studies, testosterone replacement therapy increases cardiovascular disease risks (Miner et al., 2014; Anaissie et al., 2017), but the effect sizes are too small to be convincing. More importantly, such studies cannot exclude the possibility that testosterone can reduce blood pressure for a subpopulation of males with disease-associated variants in molecules of the EPHB6 signaling pathway (including EPHB6 and CHRNA3) but that such a subpopulation, being a minor one, is obscured by others in statistical analysis in studies without stratification based on testosterone levels.

Our study with stratification of testosterone levels has enhanced the association of variants of molecules in the EPHB6 signaling pathway with hypertension risks and revealed the association of a CHRNA3 variant with hypertension risks. Our results further suggest that for the subpopulation carrying the *rs3743706* SNV, testosterone replacement therapy could be a personalized treatment for the cause of their hypertension. Although testosterone treatment might raise the concern of increased prostate cancer risks, this could be mitigated by routine examination of the patients for elevated serum prostate-specific antigen (PSA) levels. Moreover, a meta-analysis of testosterone replacement therapy outcome has shown no evidence of increased prostate cancer risks and no significant increase in PSA levels (Kang and Li, 2015).

AChR function, which is affected by its assembly, cycling, and clustering in addition to its opening, is known to be modulated by its associated molecules (Christianson and Green, 2004; Dobbins et al., 2008; Jones et al., 2010; Baier et al., 2011). We used three different experimental approaches (i.e., immunoprecipitation, colocalization, and FRET) to show that EPHB6 physically interacted with CHRNA3 in AGCCs. This provides a molecular basis for EPHB6 to function as a modulator of nAChR. AChR opening is the very first triggering event leading to a subsequent large Ca^2+^ influx, which is required for proper adrenaline secretion. Therefore, the physical interaction between EPHB6 and a dominant AChR subunit CHRNA3 implies the possibility that EPHB6 modulates AChR opening and hence the subsequent Ca^2+^ influx that controls adrenaline secretion.

To demonstrate that such interaction has functional relevance, we need to prove experimentally that KO AGCCs have reduced initial Ca^2+^/N^+^ influx through nAChR upon acetylcholine stimulation. We spent significant efforts to demonstrate this but were not able to succeed due to the technical difficulties of patching primary AGCCs. We also need to test in human patients with adequate testosterone levels but with loss-of-function variants of EPHB6 and/or its downstream signaling molecules whether less AGCC-derived catecholamines are produced according to 24-hour urine tests.

We previously demonstrated that EPHB6 KO in castrated mice led to hypertension (Luo et al., 2012). This prior knowledge plus our current findings from the human genetic study and mouse AGCC experiments allow us to propose the following model for the implication of the regulation of AGCC function by CHRNA3 and EPHB6 in human hypertension pathogenesis.

EPHB6 and molecules in its signaling pathways (e.g., CHRNA3) normally have a positive effect on catecholamine secretion by AGCCs. The results of our current study suggest that such a positive effect could be exerted by two different mechanisms. (1) A proper expression or function of molecules (e.g., CHRNA3) in the EPHB6 signaling pathway; or (2) an appropriate direct interaction at the protein level between EPHB6 and its signaling molecules (e.g., the physical interaction between EPHB6 and CHRNA3). Compromises in either of these two mechanisms will reduce catecholamine secretion by AGCCs, and such reduction has a protective effect against hypertension. The significant variant found in the CHRNA3 gene is related to mechanism 1. The physical interaction between EPHB6 and CHRNA3 in AGCCs implicates mechanism 2. Such a protective effect depends on the presence of sufficient testosterone, and hence only occurs in testosterone-sufficient males (Wang et al., 2018). At the same time, EPHB6 has other targets (e.g., vascular smooth muscle cells), and EPHB6 KO results in increased blood vessel contractility (Luo et al., 2012). Although we can only speculate at this time, the deleterious CHRNA3 variant might also have other targets leading to increased blood pressure. As a result, the protective effect due to the reduced catecholamine secretion is neutralized by the above-described blood increasing effects. Hence, males with deleterious variants in EPHB6 or its signaling molecules (e.g., CHRNA3) have normal BP. However, when their testosterone level is decreased due to either old ages or some pathological conditions, the protective effect due to reduced catecholamine secretion is lost. This is evidenced in our previous report that the castrated EPHB6 KO mice presented hypertension (Luo et al., 2012). Consistent with this hypothesis, we demonstrated that hypogonadic patients with deleterious CHRNA3 variants had increased hypertension risks according to our human genetic study.

Molecules in the EPHB6 signaling pathway (including EPHB6, EFNBs, AChR, BK, etc.) are also expressed in catecholamine-producing neuronal cells in the nervous system. Whether their sequence variants alone or in concert with sex hormones compromise catecholamine release and consequently modify the pathophysiology of diseases caused by dysfunctional catecholamine secretion (e.g., Parkinson’s disease) is an area worth investigating.

## Data Availability Statement

The raw data supporting the conclusion of this article will be made available by the authors, without undue reservation.

## Ethics Statement

The studies involving human participants were reviewed and approved by Ethics Committee of the First Affiliated Hospital of Zhejiang University (No. 2013-145). The patients/participants provided their written informed consent to participate in this study. The animal study was reviewed and approved by Animal Protection Committee of CHUM Research Center. Written informed consent was obtained from the owners for the participation of their animals in this study.

## Author Contributions

JW, HL, S-JH, and J-ZS generated the concept and initiated this project. TW, YW, WS, B-QZ, Y-MY, H-DW, Z-XX, JW, J-ZS, S-JH, JL, and RB conducted the experiments. JR and FM-B performed the genetic data analysis. YW and WS analyzed the animal data. TW, JR, YW, WS, HL, and JW drafted the manuscript. All authors contributed to the article and approved the submitted version.

## Conflict of Interest

The authors declare that the research was conducted in the absence of any commercial or financial relationships that could be construed as a potential conflict of interest.

## Abbreviations

ACh: acetylcholine
AChR: acetylcholine receptors
AGCCs: adrenal gland chromaffin cells
BK channel: large-conductance calcium-activated potassium channel
BMI: body mass index
C.H.B.: Han Chinese in Beijing
CHRNA3: nicotinic acetylcholine receptor alpha 3 subunit
EFNs: ephrins
EPHs: erythropoietin-producing hepatocellular kinases
KO: knockout
LD: linkage-disequilibrium
mAChR: muscarinic AChR
PSA: prostate-specific antigen
SNVs: single-nucleotide variants
VSMC: vascular smooth muscle cell
WT: wild type.
