# Supplementary material for: A Variant in the Nicotinic Acetylcholine Receptor Alpha 3 Subunit Gene Is Associated With Hypertension Risks in Hypogonadic Patients
Source: Front Genet. 2020 Nov 27;11:539862. doi: 10.3389/fgene.2020.539862 (PMC7728919; doi:10.3389/fgene.2020.539862)
Supplement: Supplementary file 1 [file Table_1.DOCX]

**Supplementary data**

***Supplementary Tables***

Supplementary tables 1-3 were previously published in Scientific Reports 8:14497, 2018. They are presented here upon the requests of reviewers for the convenience of the readers.

*Supplementary Table 1. Systolic and diastolic BP of cases and controls*

| Blood Pressure (BP) | Hypertensive Samples (Cases) | | | Normotensive Samples (Controls) | | | *P*-value for Difference of Means (*t*-test) |
| --- | --- | --- | --- | --- | --- | --- | --- |
|  | N | Mean | Standard Error of Mean | N | Mean | Standard Error of Mean |  |
| Systolic BP (mm Hg) | 750 | 125.42 | 0.54 | 750 | 116.6 | 0.43 | 2.2 X 10^-16^ |
| Diastolic BP (mm Hg) | 750 | 76.45 | 0.36 | 750 | 72.06 | 0.32 | 2.2 X 10^-16^ |

N: number of individuals in the group

*Supplementary Table 2. Differences in potential covariate phenotype parameters that could be implicated in causing hypertension*

| Parameter | Hypertensive Samples (Cases) | | | Normotensive Samples (Controls) | | | *P*-values for Difference between Case and Control Status |
| --- | --- | --- | --- | --- | --- | --- | --- |
|  | N | Mean | Standard Error of Mean | N | Mean | Standard Error of Mean |  |
| Age (years) | 750 | 58.80 | 0.26 | 750 | 57.66 | 0.36 | 1.04 X 10^-2^ |
| Plasma Testosterone Levels (ng/dL) | 750 | 259.76 | 2.16 | 750 | 264.75 | 2.24 | ns |
| Heart Rate (beats per minute) | 748 | 69.84 | 0.33 | 748 | 71.36 | 0.34 | 1.80 X 10^-3^ |
| Body Mass Index ( kg/m^2^) | 722 | 25.94 | 0.12 | 705 | 24.71 | 0.12 | 1.91 X 10^-12^ |
| Serum Uric Acid ( μmol/L) | 736 | 371.69 | 3.55 | 737 | 352.76 | 3.55 | 2.0 X 10^-4^ |
| Number of Smokers/Number of the individuals of the group (%) |  | 487/750 (64.9) |  |  | 468/750 (62.4) |  | ns |

ns: not statistically significant at *p*=0.05.

*P*-values were determined by simple logistic regression of individual parameters against case versus control status.

*S.-Table 3. Significance of parameters used as covariates in a stepwise multiple regression model*

| Parameter | Coefficient | Std. Error | Z-score | *P*-value |
| --- | --- | --- | --- | --- |
| (Intercept) | -3.65882 | 0.882338 | -4.15 | 3.37 X 10^-05^ |
| Age | 0.02668 | 0.006828 | 3.91 | 9.32 X 10^-05^ |
| Testosterone Levels | -0.00183 | 0.000937 | -1.95 | 5.06 X 10^-02^ |
| Heart Rate | -0.01937 | 0.006275 | -3.09 | 2.03 X 10^-03^ |
| Body Mass Index | 0.13036 | 0.018895 | 6.90 | 5.23 X 10^-12^ |
| Serum Uric Acid | 0.00185 | 0.000595 | 3.11 | 1.85 10^-03^ |


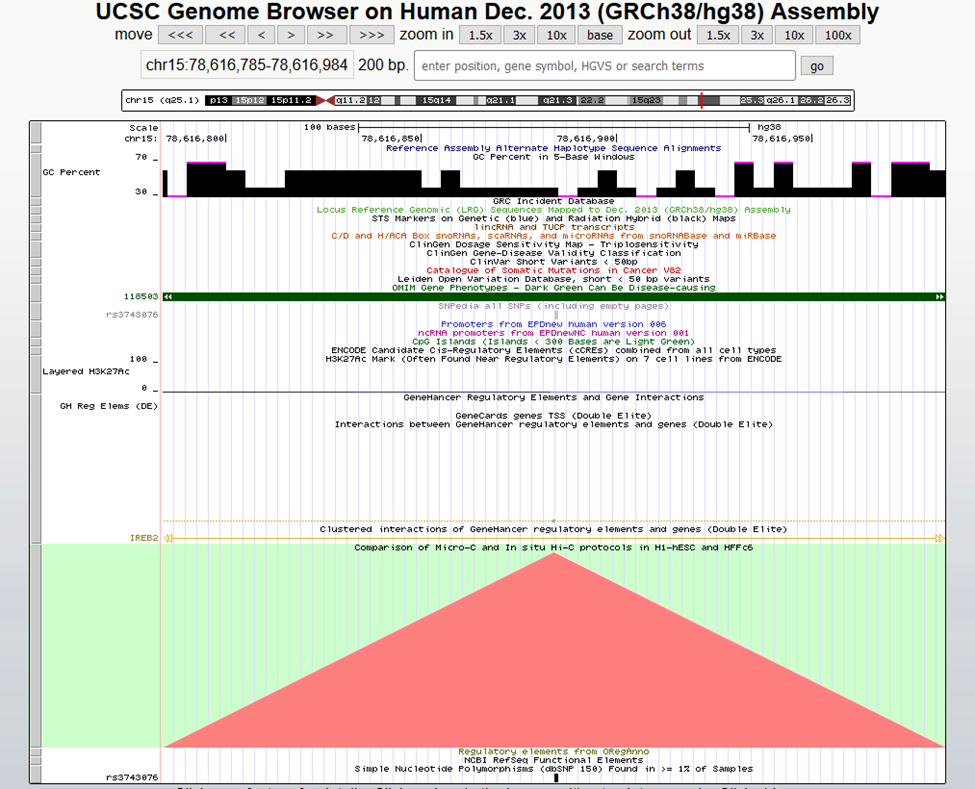
Supplementary Figure

*Supplementary Figure 1. Analysis of 200-bp region surrounding rs3743076 for regulatory motifs*

The genomic sequence from 100-bp upstream to 100-bp downstream of SNV rs374076 (Chr15:78,616,885; hg38.p12) was analyzed for the presence of possible regulatory elements using UCSC Genome Browser (EPDnew, EPDnewNC, ENCODE Candidate Cis-Regulatory Elements (cCREs), GeneHancer, CpG islands, H3K27Ac Mark, ORegAnno, NCBI RefSeq Functional Elements, Double Elite, Hi-C and Micro-C). No conserved enhancer/repressor motifs or transcription factor-binding sites were found in this region.
